# Preoperative prevalence of deep vein thrombosis in patients scheduled to have surgery for degenerative musculoskeletal disorders

**DOI:** 10.1186/s12891-021-04405-3

**Published:** 2021-06-04

**Authors:** Keigo Sato, Hideki Date, Takehiro Michikawa, Mitsuhiro Morita, Kazue Hayakawa, Shinjiro Kaneko, Nobuyuki Fujita

**Affiliations:** 1 Department of Orthopaedic Surgery, School of Medicine, Fujita Health University, 1-98 Dengakugakubo, Kutsukake-cho, Aichi Toyoake, Japan; 2 Department of Environmental and Occupational Health, School of Medicine, Toho University, Tokyo, Japan; 3 Department of Spine and Spinal Cord Surgery, Fujita Health University, Aichi Toyoake, Japan

**Keywords:** Pulmonary thromboembolism, Deep venous thrombosis, Venous thromboembolism, D-dimer, Degenerative musculoskeletal disorders, Osteoarthritis, Degenerative spinal disorders, Elective surgery

## Abstract

**Background:**

Although the incidence of symptomatic pulmonary thromboembolism after elective surgery for degenerative musculoskeletal disorders is comparatively low, it is extremely detrimental to both patients and health-care providers. Therefore, its prevention is mandatory. We aimed to perform a cross-sectional analysis of deep venous thrombosis (DVT) before elective surgery for degenerative musculoskeletal disorders, including total knee arthroplasty (TKA), total hip arthroplasty (THA), and spinal surgery, and identify the factors associated with the incidence of preoperative DVT.

**Methods:**

The clinical data of patients aged ≥ 30 years who underwent TKA or THA, and spine surgery for lumbar or cervical degenerative disorders at our institution were retrospectively collected. D-dimer levels were measured preoperatively in all the patients scheduled for surgery. For the patients with D-dimer levels ≥ 1 µg/mL or who were determined by their physicians to be at high risk of DVT, the lower extremity vein was preoperatively examined for DVT on ultrasonography.

**Results:**

Overall, we retrospectively evaluated 1236 consecutive patients, including 701 men and 535 women. Of the patients, 431 and 805 had D-dimer levels ≥ 1 and < 1 µg/mL, respectively. Of 683 patients who underwent lower extremity ultrasonography, 92 had proximal (n = 7) and distal types (n = 85) of DVT. The preoperative prevalence of DVT was 7.4 %. No patient had the incidence of postoperative symptomatic venous thromboembolism. A multivariate analysis revealed that age ≥ 80 years (odds ratio [OR], 95 % confidence interval [CI]: 2.8, 1.1–7.3), knee surgery (2.1, 1.1–4.0), American Society of Anesthesiologists (ASA) grade 2 (2.8, 1.2–6.8), ASA grades 3 or 4 (3.1, 1.0–9.4), and malignancy (1.9, 1.1–3.2) were significantly associated with DVT incidence.

**Conclusions:**

This is the first study to conduct a cross-sectional analysis of preoperative DVT data of patients scheduled for elective surgery for degenerative musculoskeletal disorders. Although whether screening for preoperative DVT is needed to prevent postoperative symptomatic pulmonary thromboembolism remains to be clarified, our data suggested that DVT should be noted before surgery in the patients with advanced age, knee surgery, high ASA physical status, and malignancy.

## Background

The number of patients with degenerative musculoskeletal disorders, including knee or hip osteoarthritis and degenerative spinal disorders, is increasing worldwide as the global population ages, and the financial and social costs associated with these conditions are also increasing in several countries [1, 2]. The therapeutic outcomes of total knee arthroplasty (TKA), total hip arthroplasty (THA), and spinal surgery are generally favorable. However, these procedures are associated with serious postoperative complications such as surgical site infection, postoperative hematoma, implant breakage, or even symptomatic pulmonary thromboembolism (PTE) [3–5].

According to the American Academy of Orthopedic Surgeons guidelines [3], the incidence of symptomatic PTE after TKA and THA is approximately 0.4 %, and this complication is fatal in approximately 10 % of the cases. The incidence of symptomatic PTE in spinal surgery is even lower, making it an extremely rare complication. However, symptomatic PTE often leads to sudden postoperative death. As its incidence is extremely detrimental to the patient and health-care providers alike, its prevention is mandatory. Therefore, the following practices are observed in clinical practice to prevent the occurrence of symptomatic PTE: screening for deep venous thrombosis (DVT), perioperative use of compression stockings or an intermittent pneumatic compression device, early postoperative active or passive motion of the knee or hip joint, early mobilization and ambulation, and prophylactic anticoagulant administration [3]. The reported risk factors of symptomatic VTE after surgery for degenerative musculoskeletal disorders include prior occurrence of symptomatic VTE, obesity, advanced age, and paralysis of the legs [3, 6, 7]. Although no current clear evidence supports the usefulness of preoperative screening for DVT by means of D-dimer measurement and ultrasonography for decreasing the incidence of symptomatic VTE, in our institution, we measure preoperative D-dimer level not only for trauma patients but also for all patients scheduled to undergo an elective surgery for a degenerative musculoskeletal disorder. If the level exceeds the reference value, we perform DVT screening by ultrasonography. Previous studies that assessed the incidence of preoperative DVT in patients with degenerative musculoskeletal disorders focused on patients undergoing a single type of surgery, including TKA, THA, or spinal surgery [8–16]. To the best of our knowledge, few single cross-sectional studies have considered all these surgeries in the assessment of the preoperative prevalence of DVT. Therefore, in this study, we aimed to perform a cross-sectional analysis of the incidence of DVT before elective surgery for degenerative musculoskeletal disorders in our institution and to identify the factors associated with the preoperative DVT.

## Methods

### Study participants

**Fig. 1:**
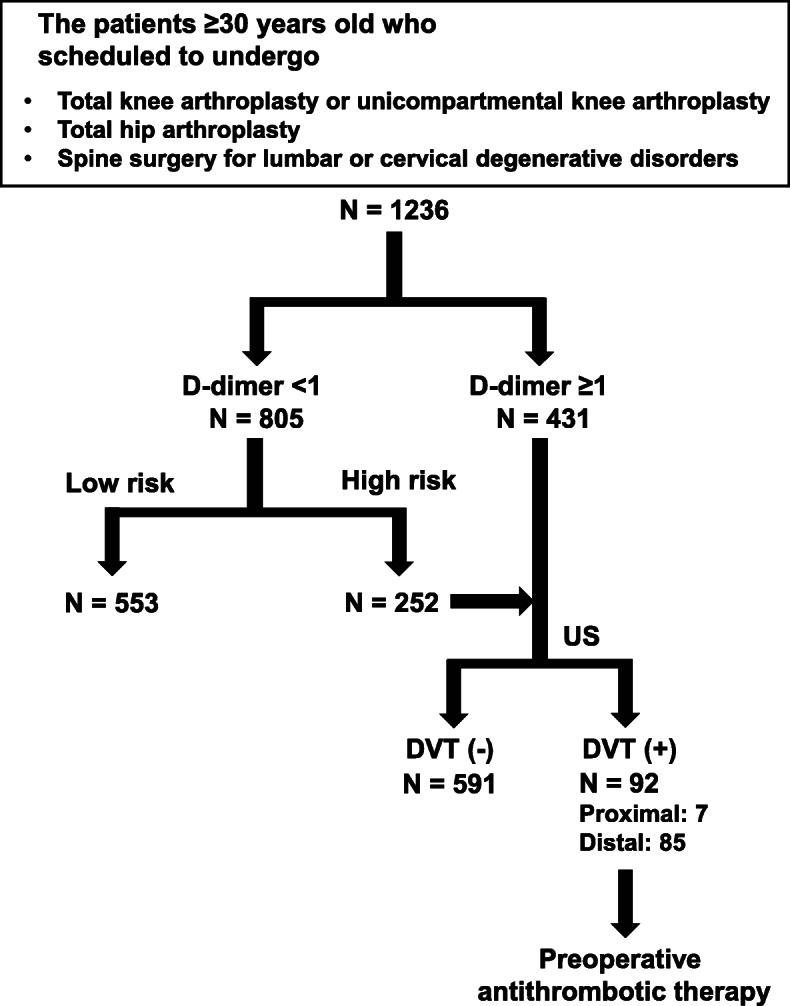
Flowchart of the screening for preoperative deep vein thrombosis (DVT). US, ultrasonography

The clinical data of patients aged ≥ 30 years who underwent TKA or unicompartmental knee arthroplasty (UKA), THA, and decompression and/or fixation spine surgery for lumbar or cervical degenerative disorders at our institution between January 2018 and December 2019 were retrospectively collected. TKA or UKA was performed for osteoarthritis, rheumatoid arthritis, and idiopathic osteonecrosis of the knee. THA was performed for osteoarthritis and rheumatoid arthritis of the hip and osteonecrosis of the femoral head. Spine surgery included both the anterior and posterior approaches. In our institution, D-dimer level in peripheral blood was measured preoperatively in all the patients scheduled for an orthopedic surgery (Fig. 1). For the patients with D-dimer levels ≥ 1 µg/mL or who were determined by the subjective judgment of physicians to be at high risk of DVT, the lower extremity vein was preoperatively examined for DVT on ultrasonography (Fig. 1). The value of D-dimer was converted by DD units in the present study. All the patients without preoperative DVT were fitted with both compression stockings and an intermittent pneumatic compression device in the perioperative period, and left bed and started walking on the first postoperative day. Meanwhile, in the patients with preoperative DVT, the protocol with compression stockings and an intermittent pneumatic compression device in the perioperative period was modified for each patient. All the patients with preoperative DVT received preoperative antithrombotic therapy including heparinization and/or oral administration of active factor Xa inhibitors. The choice of treatment, dose, and duration were determined by each patient’s physician. The patients with TKA/UKA and THA started continuous passive motion of the knee and hip joints on the first postoperative day, and oral administration of active factor Xa inhibitors for 7 days from the second postoperative day. Meanwhile, the patients with spinal surgery did not have postoperative antithrombotic therapy for avoiding spinal epidural hematoma. No patient was preoperatively placed with inferior vena cava filter.

### Ethics approval and consent to participate

 Ethical approval for this study was granted by Fujita Health University Ethics Committee (approval No. HM20-055). The informed consent was waived by the Fujita Health University Ethics Committee. Fujita Health University Ethics Committee also approved the opt-out consent process, meaning that all eligible people were included in this study unless they contacted us to opt out. No administrative permissions and/or licenses were required to access the clinical/personal patient data used in this research. All methods in the study were carried out in accordance with the Helsinki guidelines and declaration or any other relevant guidelines.

### Diagnosis of DVT

Linear and convex probes (Canon, Tokyo, Japan) were used to perform pulse Doppler ultrasonographic examinations of the lower extremities of the patients. The transducer frequency was set from 7.5 MHz to 12 MHz. The diagnostic criteria for DVT are a filling defect of the cavity, incompressibility of the vein, and lack of Doppler signal. Routine scanning for vein thrombosis was performed for the common femoral, femoral, popliteal, posterior tibial, peroneal, soleal, and sural veins of the bilateral lower extremities. In the case of common femoral, femoral, and popliteal veins, it was diagnosed as the proximal type. Meanwhile, in the case of posterior tibial, peroneal, soleal, and sural veins, it was diagnosed as the distal type.

### Data collection

The following data were evaluated: age, body mass index (BMI), sex, surgical site, American Society of Anesthesiologists (ASA) physical status grade, and medical history, including hypertension, hyperlipidemia, diabetes, stroke, malignancy, and smoking.

### Statistical analyses

Data are presented as mean ± standard deviation. The Mann-Whitney *U* test, Pearson chi-square test, or analysis of variance was used for the statistical analysis. Multiple comparisons among the four groups, including the knee, hip neck, and low back, were performed using the Dunnett test. To examine the independent associations of DVT, we constructed a logistic regression model that included age, sex, BMI, surgical site, ASA grade, hypertension, hyperlipidemia diabetes, stroke, and malignancy, and the estimated odds ratios (ORs) and 95 % confidence intervals (CIs) for DVT. Logistic regression was performed by using the Stata 14 software (Stata Corporation, College Station, TX). *P* values < 0.05 were considered as indicating statistical significance.

## Results

**Fig. 2:**
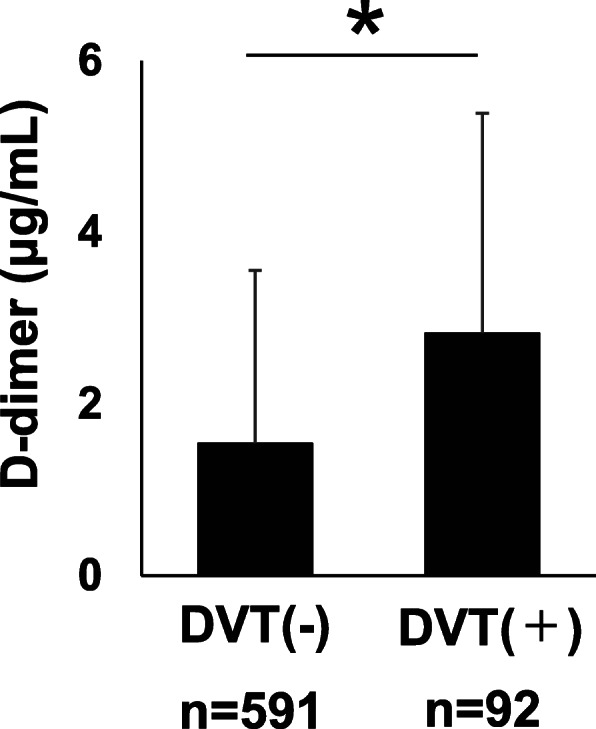
Comparison of the mean D-dimer level in the patients with and those without DVT. Data are presented as mean ± SD. *p < 0.05

 Overall, we retrospectively reviewed 1236 consecutive patients (701 men and 535 women) in the present study. The patients’ baseline characteristics, including age, sex, BMI, surgical site, and medical history, are summarized in Table 1. Figure 1 presents the flowchart for the examination for DVT. Of the patients, 431 had D-dimer levels ≥ 1 µg/mL and 805 had D-dimer levels < 1 µg/mL. The patients with D-dimer levels ≥ 1 (*n* = 431) and < 1 µg/mL, who were judged by the physician to have a high risk of DVT (*n* = 252), underwent DVT examination of the lower extremity veins on ultrasonography. Of the 683 patients who underwent lower extremity ultrasonography, 92 had proximal (*n* = 7) and distal types (*n* = 85) of DVT. The preoperative prevalence of DVT was 7.4 %. In one patient with preoperative leg edema, surgery was delayed (*n* = 1). Consequently, no patient had the incidence of postoperative symptomatic VTE. Figure 2 showed the mean D-dimer level in the patients with and without DVT. We confirmed that the mean D-dimer level in the patients with DVT was significantly higher than that in the patients without DVT (Fig. 2). The features of DVT were summarized in Table 2.

**Table 1:** Baseline characteristics (*n* = 1236)

Age (y)	< 50	134 (10.8 %)
	50–59	166 (13.4 %)
	60–69	289 (23.4 %)
	70–79	467 (37.8 %)
	≥ 80	180 (14.6 %)
Sex	Men	701 (56.7 %)
	Women	535 (43.3 %)
Group	knee	211 (17.1 %)
	hip	328 (26.5 %)
	neck	174 (14.1 %)
	low back	523 (42.3 %)
BMI (kg/m^2^)	< 18.5	53 (4.3 %)
	18.5–24.9	674 (54.5 %)
	≥ 25.0	509 (41.2 %)
Hypertension	643 (52.0 %)	
Hyperlipidemia	300 (24.3 %)	
Diabetes	262 (21.2 %)	
Stroke	82 (6.6 %)	
Malignancy	146 (11.8 %)	
Smoking	523 (42.3 %)	

*BMI* Body mass index

**Table 2:** Characteristics of deep venous thrombosis (*n*=92)

Location	Proximal	7
	Distal	85
Unilateral or Bilateral	Unilateral	78
	Bilateral	14
Fresh	Yes	85
	No	7
Symptomatic	Yes	1 (leg edema)
	No	91

Next, we independently examined the mean D-dimer level and the prevalence of DVT at four surgical sites, namely the knee, hip, neck, and low back (Table 3). The mean D-dimer level and incidence of DVT in the knee group were significantly higher than those in the other groups (Table 3). The mean age and BMI associated with the development of DVT [3, 6] were also significantly higher in the knee group than in the other groups (Table 3).

**Table 3:** Comparison among different surgical sites

	knee (211)	hip (328)	neck (174)	low back (523)	P* value
Age (y)	74.0 ± 8.6	66.6 ± 11.7* *	63.4 ± 13.5* *	66.4 ± 14.3* *	<0.01
Male/ Female	50 / 161	53 / 275	126 / 48	306 / 217	<0.01
BMI (kg/m^2^)	26.2 ± 4.6	24.0 ± 3.9* *	24.0 ± 3.5* *	24.6 ± 4.2* *	<0.01
D-dimer (μg/mL)	1.85 ± 2.99	1.32 ± 1.38* *	0.86 ± 10.08* *	1.00 ± 1.40* *	<0.01
DVT (%)	14.7 % (31)	6.1% (20)	4.0% (7)	6.3 % (34)	< 0.01

* ANOVA or x^2^ test.

* * *P* < 0.05 (Dunnett test; reference = group knee).

*BMI* Body mass index

In the uni-variable analysis, DVT was statistical significantly associated with age, sex, surgical site, smoking, ASA, hypertension, and malignancy (Table 4). We constructed a multivariate model included all these variables, and found that age ≥ 80 years (OR, 2.8; 95 % CI, 1.1–7.3; reference, 50–59 years), knee surgery (OR, 2.1; 95 % CI, 1.1–4.0; reference, hip surgery), ASA grade 2 (OR, 2.8; 95 % CI, 1.2–6.8; reference, ASA grade 1), ASA grade 3 or 4 (OR, 3.1; 95 % CI, 1.0–9.4; reference, ASA grade 1), and malignancy (OR, 1.9; 95 % CI, 1.1–3.2) were significantly associated with DVT (Table 4).

**Table 4:** Logistic regression analysis of DVT

		Number of patients	Number of DVT	Rate of DVT (%)	Uni-variable analysis				Multi-variable analysis			
					OR	95% CI		*p*-value	OR	95% CI		*p*-value
Age (y)	<50	134	1	0.8	0.20	0.02	1.69	0.14	0.24	0.03	2.07	0.20
	50-59	166	6	3.6	reference							
	60-69	289	19	6.6	1.88	0.73	4.80	0.19	1.46	0.56	3.81	0.44
	70-79	467	40	8.6	2.50	1.04	6.01	0.04	1.71	0.69	4.23	0.25
	>=80	180	26	14.4	4.50	1.80	11.24	< 0.01	2.83	1.09	7.31	0.03
Sex	Men	535	25	4.7	reference							
	Women	701	67	9.6	2.16	1.34	3.46	< 0.001	1.82	0.97	3.39	0.06
Group	knee	211	31	14.7	2.65	1.47	4.79	0.00	2.12	1.13	3.96	0.02
	hip	328	20	6.1	reference							
	neck	174	7	4.0	0.65	0.27	1.56	0.33	0.98	0.38	2.49	0.96
	low back	523	34	6.5	1.07	0.61	1.89	0.81	1.27	0.68	2.38	0.45
BMI (kg/m2)	<18.5	53	7	13.2	1.78	0.76	4.13	0.18				
	18.5-24.9	674	53	7.9	reference							
	>=25.0	509	32	6.3	0.78	0.50	1.23	0.29				
Smoking	No	713	63	8.8	reference							
	Yes	523	29	5.5	0.60	0.38	0.95	0.03	1.08	0.60	1.92	0.81
ASA	1	253	6	2.4	reference							
	2	856	73	8.5	3.84	1.65	8.93	0.00	2.80	1.15	6.83	0.02
	3, 4	104	10	9.6	4.38	1.55	12.39	0.01	3.13	1.04	9.41	0.04
Hypertension	No	593	35	5.9	reference							
	Yes	643	57	8.9	1.55	1.00	2.40	0.05	0.87	0.54	1.41	0.57
Hyperlipidemia	No	936	67	7.2	reference							
	Yes	300	25	8.3	1.18	0.73	1.90	0.50				
Diabetes	No	974	76	7.8	reference							
	Yes	262	16	6.1	0.77	0.44	1.34	0.35				
Stroke	No	1,154	86	7.5	reference							
	Yes	82	6	7.3	0.98	0.42	2.32	0.96				
Malignancy	No	1,090	73	6.7	reference							
	Yes	146	19	13.0	2.08	1.22	3.57	0.01	1.85	1.06	3.23	0.03

*BMI* Body mass index

## Discussion

To the best of our knowledge, this is the first study to conduct a cross-sectional analysis of preoperative DVT data of patients scheduled for elective surgery for degenerative musculoskeletal disorders. Our data showed that the preoperative prevalence of DVT in the patients scheduled to have surgery for degenerative musculoskeletal disorders was 7.4 %. In addition, our analysis revealed that the factors associated with preoperative DVT were advanced age, knee surgery, high ASA physical status, and malignancy.

According to the American College of Chest Physicians’ evidence-based clinical practice guideline (ACCP), TKA and THA themselves are risk factors of DVT [4]. In the present study, 6.1 % of the patients scheduled for THA had DVT, even before the surgery. This proportion is consistent with those reported in previous studies (5.2–12.3 %) [8–10]. Although DVT screening methods, either ultrasonography or computed tomography, differ among studies, previous studies reported the discrepancies in the frequency of preoperative DVT in TKA (2.6–11.7 %) as well as THA [11–13]. The prevalence of preoperative DVT in TKA in the present study was 14.7 %, which is higher than those in previous studies. Owing to issues on radiation exposure with CT [17], ultrasonography will probably be the main method used in screening for preoperative DVT. Furthermore, the advancements in ultrasonography technology could continuously increase the detection rate of preoperative DVT [18]. Although D-dimer has the high rate of false positives for detecting DVT [19], we measured D-dimer levels in all patients as a simple screening tool for DVT in the present study. As expected, our data showed that the D-dimer levels were significantly higher in the patients with than in those without preoperative DVT. The cutoff D-dimer level in the first DVT screening was 1.0 µg/mL in the present study, although the ideal value has yet to be determined and is a topic for future research.

The multivariate analysis in the present study revealed that knee surgery with TKA or UKA is one of the factors associated with the preoperative DVT. Besides knee surgery, the other factors found to be associated with preoperative DVT in our analysis were advanced age, ASA physical status, and malignancy, which were consistent with the previously reported risk factors of postoperative VTE [3, 20]. In patients with knee osteoarthritis, restricted knee flexion and extension due to knee pain or limited joint range of motion may result in DVT prevalence in the veins of the lower extremities. In addition, Baker’s cyst, commonly associated with knee osteoarthritis, has been reported to compress the neurovascular bundle in the popliteal fossa [21]. Therefore, cysts around the knee joint that are associated with knee osteoarthritis may increase the frequency of DVT. In the future, the relationship between knee osteoarthritis severity and incidence of DVT should be examined.

According to the ACCP, the risk of VTE is considered moderate in spinal surgeries for malignant diseases but relatively low in elective surgeries for degenerative spinal disorders [4]. Recently, Winther et al. reported that the incidence of symptomatic VTE in elective surgeries for degenerative spinal disorders was 0.2 % [14]. Meanwhile, Takahashi et al. retrospectively examined elective spinal surgeries in a group of patients who underwent the procedure to prevent VTE and in a group who did not [22]. They found that the incidence of symptomatic PTE was significantly lower in the preventive VTE group [22]. Therefore, although the frequency of elective surgeries for degenerative spinal disorders is low, preventing VTE is observed to be important, as well as performing TKA and THA. According to Liu et al., the incidence of preoperative DVT in patients with cervical spondylotic myelopathy scheduled for spinal surgery was 4 %, which is similar to the results of this study [15]. Meanwhile, Yamasaki et al. found that the incidence of preoperative DVT in elective lumbar spine surgeries was 7.7 % when the vertebral body fracture group was excluded [16], which is also similar to the lumbar surgery data from the present study. As the population ages, patients undergoing elective surgeries for degenerative spinal disorders will be older, their ASA physical status will be higher, and majority of them will have malignancies. Therefore, the risk of VTE incidence is also expected to continuously increase, making symptomatic VTE prevention in patients undergoing elective surgeries for degenerative spinal disorders even more important.

This study has several limitations. First, our data were retrospectively collected from a limited number of patients in a single institution. Second, a selection bias exists in this study because ultrasonography was added as a screening procedure in the patients with D-dimer levels < 1 µg/mL, whose attending physicians made a subjective assessment of high DVT risk. An objective evaluation including the Caprini scale was not performed when clarifying patients with high DVT risk. Third, because the occurring of DVT does not mean episode of a fatal PTE, the present study did not have a screen of PTE. Fourth, whether screening for preoperative DVT is needed to prevent postoperative symptomatic VTE remains to be clarified. Chang et al. reported that routine preoperative DVT evaluation is probably not necessary [23]. However, if patients scheduled for elective surgery for degenerative musculoskeletal disorders have DVT before the surgery, the risks of thrombus extension and fatal PTE could be higher. Whether DVT screening through preoperative D-dimer measurements or ultrasonography can reduce the incidence of symptomatic VTE remains to be verified using large-scale data. Nevertheless, our cross-sectional analysis clearly identified the factors associated with preoperative DVT in patients with degenerative musculoskeletal disorders.

## Conclusions

We conducted a cross-sectional analysis of the prevalence of preoperative DVT in patients scheduled to undergo elective surgery for degenerative musculoskeletal disorders. Our data suggest that DVT should be noted before surgery in patients with advanced age, knee surgery, high ASA physical status, and malignancy.

## Data Availability

The datasets generated and/or analysed during the current study are not publicly available due to limitations of ethical approval involving the patient data and anonymity but are available from the corresponding author on reasonable request.

## Abbreviations

TKA: Total knee arthroplasty
THA: Total hip arthroplasty
PTE: Pulmonary thromboembolism
DVT: Deep vein thrombosis
VTE: Venous thromboembolism
